# Machine learning for multiple sclerosis classification and disability prediction using clinical and MRI data

**DOI:** 10.3389/frai.2026.1792870

**Published:** 2026-04-10

**Authors:** Paola Valsasina, Loredana Storelli, Nicolò Tedone, Patrizia Pantano, Claudia Piervincenzi, Silvia Tommasin, Antonio Gallo, Manuela Altieri, Gianpaolo Maggi, Nicola De Stefano, Alessia Bianchi, Marco Battaglini, Giulia Mazzetti, Elisabetta Pagani, Maria A. Rocca, Massimo Filippi

**Affiliations:** 1 Neuroimaging Research Unit, Division of Neuroscience, IRCCS San Raffaele Scientific Institute, Milan, Italy; 2 Vita-Salute San Raffaele University, Milan, Italy; 3 Department of Human Neurosciences, Sapienza University of Rome, Rome, Italy; 4 IRCCS Neuromed, Pozzilli, Italy; 5 UniCamillus - International Medical University in Rome, Rome, Italy; 6 First Division of Neurology and Neurophysiopathology, AOU Luigi Vanvitelli, Naples, Italy; 7 Department of Advanced Medical and Surgical Sciences, University of Campania “Luigi Vanvitelli”, Naples, Italy; 8 Department of Medicine, Surgery and Neuroscience, University of Siena, Siena, Italy; 9 SIENA Imaging SRL, Siena, Italy; 10 Neurology Unit, IRCCS San Raffaele Scientific Institute, Milan, Italy; 11 Neurorehabilitation Unit, IRCCS San Raffaele Scientific Institute, Milan, Italy; 12 Neurophysiology Service, IRCCS San Raffaele Scientific Institute, Milan, Italy

**Keywords:** artificial intelligence, classification, machine learning, MRI, multiple sclerosis

## Abstract

**Introduction:**

Multiple sclerosis (MS) is a complex disease characterized by diverse clinical presentations and progression patterns. Accurate classification and prediction of disease severity are crucial for personalized treatment. We applied machine learning (ML) to demographic, clinical and MRI data to distinguish MS patients from healthy controls (HC), classify MS phenotypes and predict disability using the Expanded Disability Status Scale (EDSS) score.

**Methods:**

We included 1,554 MS patients and 520 HC from the Italian Neuroimaging Network Initiative repository, all with neurological assessment and brain T2-/3D T1-weighted MRI. Derived MRI features included total and regional T2 lesion volumes (LV), and normalized tissue volumes from cortical and subcortical grey matter (GM), white matter, cerebellum and brainstem. ML models, including support vector machines, multi-layer perceptron networks, Random Forest and Gradient Boosting were trained for classification and prediction tasks. SHAP analysis ranked the most influential variables.

**Results:**

ML models achieved 89–96% accuracy in distinguishing MS patients from HC, driven mainly by T2 LV and brainstem/cerebellar GM volumes. Relapsing *vs* progressive MS was classified with 92% accuracy, with EDSS, age, thalamic and cortical GM volumes as key predictors. EDSS prediction achieved an intra-class correlation of 0.56–0.76; most relevant contributors were T2 LV, sex, cortical/cerebellar GM and thalamic volumes.

**Discussion:**

ML models demonstrated high accuracy in detecting MS, differentiating phenotypes, and predicting disability. Integrating demographic, clinical and MRI measures emerges as an effective strategy for patients’ classification and disease severity assessment.

## 1 Introduction

Multiple sclerosis (MS) is a chronic inflammatory, demyelinating, and neurodegenerative disease of the central nervous system (CNS) leading to the accumulation of irreversible clinical disability (Filippi et al., 2018). Magnetic resonance imaging (MRI) has become one of the most important paraclinical tools in the management of MS, owing to its high sensitivity in detecting focal lesions and its ability to capture the diverse pathological substrates of the disease, including those related to neurodegeneration. Beyond its diagnostic role, MRI plays a crucial part in elucidating disease pathophysiology, monitoring disease progression, and assessing treatment response (Rocca et al., 2024). With the wider availability of MRI and continuous technological advances, the volume of MRI data collected from MS patients has increased substantially, offering novel opportunities for advanced computational analyses (Vrenken et al., 2021).

In recent years, artificial intelligence (AI) has experienced an extremely rapid development, and a growing number of applications are emerging across the medical field. Recent progress in machine learning (ML) and deep learning (DL) methods enabled promising applications in MS research: several AI algorithms have been applied to MRI data in MS with the main goal of producing automated lesion (Aslani et al., 2019; Preziosa et al., 2025; Valverde et al., 2017) or tissue segmentation (Bonacchi et al., 2022a; Narayana et al., 2020). However, AI technologies have also been increasingly applied to MS differential diagnosis, disease classification, prognosis, and treatment monitoring (Bonacchi et al., 2022a; Amin et al., 2024; Collorone et al., 2024).

More specifically, DL algorithms applied to MRI data have shown to be able to differentiate MS patients from healthy controls (HC) (Zhang et al., 2018); moreover, DL and ML analyses have demonstrated good diagnostic accuracy in distinguishing MS patients from other mimickers, such as neuromyelitis optica spectrum disorder (Eshaghi et al., 2016), CNS vasculitis or migraine (Rocca et al., 2021a). ML techniques applied to advanced, network-based structural and functional MRI metrics have shown a good ability to classify MS patients according to disease phenotype (Barile et al., 2022; Kocevar et al., 2016; Marzullo et al., 2019; Yamin et al., 2023), and clarifying their associations with clinical disability and cognitive impairment (Yousef et al., 2024). Another interesting topic is the application of AI to prognostic modeling. ML and DL approaches have demonstrated potential in predicting conversion from clinically isolated syndrome (CIS) to definite MS, forecasting future relapses, and identifying patients at risk of long-term disability progression (Collorone et al., 2024; Zhang et al., 2018; Campanioni et al., 2024; Pontillo et al., 2021; Taloni et al., 2022; Tommasin et al., 2021). In addition, AI has enhanced the ability to detect treatment response, with some models achieving high discriminative performance (Kanber et al., 2019).

Despite these encouraging findings, several limitations hinder AI translation into clinical practice; in particular, most of studies have been conducted with relatively small cohorts, thereby limiting generalizability and robustness (Bonacchi et al., 2022a; Amin et al., 2024; Collorone et al., 2024). MS is highly heterogeneous, and reliable classification/stratification tasks require large, diverse, and standardized datasets capturing variability across ages, phenotypes, and disease stages, a condition that can be more readily achieved within multicenter collaborations using relatively homogeneous data collection protocols. In this context, the Italian Neuroimaging Network Initiative (INNI) repository (Filippi et al., 2017) represents a unique opportunity to advance the field. Within the INNI platform, a large amount of clinical and MRI data has been collected using relatively standardized protocols, including sequences dedicated to measurement of lesion burden, global and regional brain atrophy, microstructural integrity and functional connectivity. Some of these measures have already been incorporated into AI studies, which have demonstrated that ML models are capable of predicting patients’ cognitive status (Marzi et al., 2023).

In this project, we hypothesized that developing integrated ML models incorporating demographic, clinical and MRI features may increase our ability to detect features associated with the more unfavorable aspects of MS disease, with potential implications for clinical decision-making and patient management. To verify this, we applied different ML models to multifaceted datasets, including demographic, clinical, and structural MRI variables, to test their ability models to: (i) distinguish MS patients from HC; (ii) classify patients according to disease clinical phenotype; and (iii) predict patients’ clinical disability.

## 2 Methods

### 2.1 Standard protocol approvals, registrations, and patient consents

The Institutional Ethical standards Committee on human experimentation at IRCCS Ospedale San Raffaele approved the experiment (Protocol N° PNRR-MAD-2022-12376530). Before participating in the study, all subjects provided written informed consent in accordance with the Declaration of Helsinki.

### 2.2 Study population

For this study, we retrospectively analyzed data from the INNI repository,^1^ which includes subjects enrolled at four Italian MS centers: IRCCS San Raffaele Scientific Institute (Milan), University of Campania “Luigi Vanvitelli” (Naples), Sapienza University of Rome (Rome), and University of Siena (Siena). Eligible patients met the following criteria: (1) diagnosis of CIS or clinically definite MS, according to 2017 revisions of McDonald criteria (Thompson et al., 2018); (2) age ≥18 years; (3) availability of demographic and clinical data (including clinical phenotype, disease duration, and disability); (4) availability of a brain T2-weighted scan for T2-hyperintense lesion assessment; and (5) availability of a brain 3D T1-weighted MRI for atrophy assessment.

From the INNI repository, we also retrospectively selected a pool of HC with available brain T2-weighted and 3D-T1-weighted MRI scans. In addition, HC were matched to MS patients for age, while ensuring an adequate representation of HC from each center to provide sufficient site-specific variability and to minimize center-related bias. Inclusion criteria for both MS patients and HC were also: no history of alcohol or substance abuse, no neurologic diseases (other than MS), and no psychiatric diseases.

### 2.3 Neurological assessment

Experienced neurologists, blinded to MRI results, performed a neurologic examination, including the main information about disease history (i.e., disease onset date, clinical phenotype) and rating of the Expanded Disability Status Scale (EDSS) score (Kurtzke, 1983). Information about disease-modifying treatment (DMT) were also collected.

### 2.4 MRI acquisition

Using 3.0 T scanners at all sites (Milan: Philips Achieva and Ingenia, Siena: Philips Achieva, Best, The Netherlands; Rome: Siemens Magnetom Verio, Erlangen, Germany; Naples: GE Signa HDxt and DV750, Milwaukee, USA) the following MRI scans were obtained from each study subject: (1) dual-echo (DE) turbo spin echo (TSE) or fluid-attenuated inversion recovery (FLAIR) for lesion assessment; and (2) 3D T1-weighted scan for atrophy assessment. Detailed sequence parameters implemented on each scanner are reported in Supplementary Table 1.

### 2.5 Structural MRI analysis

#### 2.5.1 Analysis of T2-hyperintense white matter (WM) lesion volume (LV)

On subjects having DE TSE or 2D FLAIR scans, T2-hyperintense lesions were segmented at each participating site using a semi-automated technique (Jim 8.0, Xinapse System, Colchester, UK),^2^ while on subjects having a 3D FLAIR acquisition, lesions were detected by using a fully automated pipeline based on a cascade of two 3D patch-wise convolutional neural networks (Valverde et al., 2017). To avoid center-dependent variability of lesion marking, all lesion masks were reviewed centrally by experts and edited (if necessary), as previously described (Filippi et al., 2017). T2 lesion volume (LV) was then calculated using the FSL toolbox.

To examine whether lesion location influences classification, we also analyzed T2 LV according to its topographical distribution. Lesion topography was defined as follows: each lesion was first identified using a MATLAB routine that labels connected components. Subsequently, the relevant tissue masks obtained from the FMRIB Software Library (FSL) SIENAx2 (Battaglini et al., 2018) (including cortical grey matter (GM), lateral ventricles, cerebellum, and brainstem) were transformed into the lesion segmentation space. Lesions located in the cerebellum and brainstem were classified as infratentorial (IT). After applying a 3 mm spherical kernel, periventricular (PV) and juxtacortical (JC) lesions were identified as those in contact with the respective dilated masks, while all remaining lesions were categorized as deep white matter (WM). For each lesion class, LV of corresponding T2-hyperintense lesions was then computed.

#### 2.5.2 Analysis of whole-brain and regional brain atrophy

3D T1-weighted sequences images underwent a pre-processing pipeline consisting of distortion correction and signal inhomogeneity correction using the N4 algorithm (Storelli et al., 2019), followed by lesion filling (Battaglini et al., 2012). Then, normalized cortical GM volume (NCGMV) and normalized WM volume (NWMV) were obtained using FSL SIENAx2 (Battaglini et al., 2018). Automatic segmentation of deep GM structures (thalamus, putamen, pallidum, caudate, amygdala and nucleus accumbens) and of hippocampus was performed using FSL FIRST (Battaglini et al., 2018); such volumes were subsequently adjusted by applying the FSL SIENAx2 scaling factor. Considering their potential relevance for classification and prediction tasks, the following measures were further considered for statistical analyses: normalized hippocampal volume (NHippV), normalized thalamic volume (NThalV), and normalized volume of other deep GM nuclei (NOtherDGMV), defined as the sum of caudate nucleus, pallidum, putamen, amygdala, and nucleus accumbens. Lastly, segmentation of GM and WM of the cerebellum, as well as segmentation of brainstem, were performed using Freesurfer 7.1,^3^ followed by AI-based editing procedures. More specifically, we used a dataset comprising 2,491 3D T1-weighted MRI scans of HC collected from 10 publicly available databases. The cerebellum and four brainstem subfields (medulla oblongata, pons, midbrain, and superior cerebellar peduncle) were segmented automatically using FreeSurfer 7.1, and the resulting labels were considered as silver-standard annotations. The dataset was divided into a training set (2087 images) and a test set (404 images), ensuring that all test subjects originated from imaging centers not represented in the training data. A dedicated U-Net–based convolutional neural network (CNN) was trained for this task. Training was performed with an initial learning rate of 0.001, adjusted by a polynomial scheduler, using a batch size of 1 over 500 epochs. The loss function combined Dice loss and cross-entropy loss to improve training stability, and optimization was performed using the Adam optimizer. The resulting DL pipeline was subsequently applied to the MRI scans included in the present study to segment cerebellar WM/GM and brainstem, and to quantify their respective volumes for further analyses. Normalized volumes of the cerebellar WM (NCerWMV), normalized volumes of cerebellar GM (NCerGMV), and normalized brainstem volume (NBrainstemV) were finally obtained by multiplying derived volumes for the FSL SIENAx2 scaling factor.

Before statistical analysis, MRI measures underwent a harmonization procedure to reduce scanner-related effects, while preserving interindividual biological variability, using the NeuroComBat package (Fortin et al., 2018). Age, sex and clinical phenotype were incorporated into the harmonization process to account for intersubject biological variability. As shown in Supplementary Figure 1, the method successfully mitigated inter-scanner variability, producing harmonized measures across the different MRI scanners.

### 2.6 Classification and prediction tasks using ML techniques

#### 2.6.1 Task description

After performing the MRI analysis described above, we investigated the ability of advanced ML methods to classify MS patients and predict their clinical disability using different techniques. The goal of the first classification task was to distinguish MS patients from HC, while the aim of the second classification task was to classify relapsing (i.e., CIS and relapsing–remitting patients) from progressive MS patients. Finally, we performed a regression task to directly predict patients’ EDSS score.

With regards to the first classification task (MS patients *vs* HC), two sensitivity analyses were also performed to verify robustness of results: (i) we restricted the analysis to early MS patients (i.e., those with a disease duration ≤5 years), to evaluate classification performance in a MS group more comparable to HC, in whom this task is more clinically relevant; (ii) we evaluated the possible effect of lesion topography by considering T2 LV divided in the anatomical regions previously described, to investigate whether specific lesion locations were more informative for classification. A third sensitivity analysis consisted in performing the main classification tasks by using MRI-derived features alone, excluding demographic and clinical variables.

#### 2.6.2 ML tools

To explore the influence of different model complexities, four supervised ML algorithms were implemented: support vector machine (SVM) (Vapnik, 1997), multi layer perceptron (MLP) (Pal and Mitra, 1992), Random Forest (Ho, 1995) and Gradient Boosting (Hastie et al., 2009) applied to both classification and regression tasks. The SVM was selected for its ability to capture both linear and non-linear relationships through kernel functions, while the MLP was included as a straightforward linear model particularly suited for high-dimensional datasets. Random Forest was selected for its robustness to noise and overfitting and its ability to handle heterogeneous biomedical features while providing feature importance estimates. Finally, Gradient Boosting method was adopted for its high predictive accuracy by sequentially improving weak learners and capturing subtle patterns in the data. Prior to model training, all features were standardized to ensure comparability across variables and to improve numerical stability. Specifically, each feature was rescaled using z-score normalization (mean = 0, standard deviation = 1) based on the training set statistics, and the same transformation was applied to the test data.

The SVM model was configured to handle class imbalance and adjusted the class weights inversely to the class frequencies in the training set. Probability estimation was enabled via Platt’s scaling to obtain calibrated class probabilities. As a second approach, MLP was implemented and trained using stochastic gradient descent, also accounting for class imbalance during optimization. In addition, a Random Forest model with class-balanced weights was employed, with hyperparameters optimized through Grid Search Cross-Validation using 3-fold cross-validation and the F1-score as the optimization metric. Finally, a Gradient Boosting model was implemented, and its hyperparameters were tuned using the same grid search cross-validation procedure.

All models were trained using a 70/30 train-test split, with 70% of the data used for model training and 30% held out as an independent test set to evaluate final performance. Within the training set, hyperparameters were optimized using an inner 3-fold cross-validation, ensuring that model selection and tuning did not include the test data. Specifically, for the SVM, an exhaustive grid search was performed over multiple kernel types (linear, RBF, polynomial, sigmoid) and associated hyperparameters, with the F1-score used as the optimization metric. Similarly, the MLP, Random Forest and Gradient Boosting hyperparameters were tuned via 3-fold cross-validation using F1-score.

Once the best hyperparameter configuration was identified for each model, the final model was retrained on the entire training set and evaluated on the held-out test set, providing unbiased performance estimates. This nested training strategy ensures model generalizability and minimizes the risk of overfitting.

All ML models were implemented in Python version 3.12.12 using the scikit-learn library.

#### 2.6.3 Model explainability

Model interpretability was assessed using the Shapley Additive explanations (SHAP) framework (Lundberg and Lee, 2017), which quantifies the marginal contribution of each feature to model predictions, providing a consistent and comparable measure of feature importance. A model-agnostic SHAP explainer was employed to ensure interpretability across different ML algorithms. A random subset of 100 instances from the scaled training data was used as background data to approximate the model’s expected output distribution, and SHAP values were computed for a stratified sample of 100 training instances to identify the features most strongly influencing model decisions.

### 2.7 Statistical analysis

Group differences in demographic and clinical variables were assessed using Chi-square tests, Mann–Whitney U tests, or Kruskall-Wallis test with *post hoc* contrasts, as appropriate. Comparisons of T2 LV and brain volumetry measures between MS patients and HC, as well as across MS phenotypes, were performed using Kruskall-Wallis test with *post hoc* contrasts. T2 LV were log-transformed prior to analysis. Statistical significance was set at *p* < 0.05. Such analyses were conducted using SPSS (IBM® SPSS® Statistics, version 29.0).

All final models (SVM, MLP, Random Forest and Gradient Boosting) were evaluated on classification and regression tasks. For classification, performance was assessed using accuracy, precision, recall, F1-score, and AUC-ROC on the validation cohort. For the prediction of EDSS, model performance was evaluated using the mean absolute error (MAE), root mean square error (RMSE), and the intra-class correlation coefficient (ICC) on the validation cohort, providing a comprehensive assessment of predictive accuracy.

## 3 Results

### 3.1 Main demographic, clinical, and structural MRI findings

The main demographic, clinical and structural MRI variables of study subjects are summarized in Table 1. Overall, there were 520 HC and 1,554 MS patients: of these, 1,143 were relapsing MS patients and 411 were progressive MS (294 [72%] secondary progressive and 117 [28%] primary progressive MS). Compared with HC, the full MS cohort showed no significant differences in mean age but a different proportion of males/females; patients also had higher T2 LV and significantly reduced normalized GM and WM volumes than HC across all examined regions (all *p* < 0.001). Considering differences between MS phenotypes, compared to relapsing MS, progressive MS patients were older (*p* < 0.001), had longer disease duration (*p* < 0.001) and higher EDSS score (*p* < 0.001). As expected, the distribution of the disease-modifying treatment (DMT) types used differed significantly between the two groups (*p* < 0.001).

**Table 1:** Main demographic, clinical and MRI variables of healthy controls (HC) and patients with multiple sclerosis (MS).

Variables	HC *N* = 520	MS patients *N* = 1,554	*p*	Relapsing MS *N* = 1,143	Progressive MS *N* = 411	*p* HC *vs* Rel MS	*p* Rel *vs* Prog MS
Site distribution (OSR/VAN/SAP/SIE)	274/125/91/30	765/300/345/144	**0.01**^**†**^	441/272/294/136	324/28/51/8	**0.001**^**†**^	**0.001**^**†**^
Mean age (IQR) [y]	41.3 (29.0;53.0)	41.7 (33.0;50.0)	0.07^*^	38.6 (30.0;46.0)	50.3 (44.0;57.0)	0.01^**^	<0.001^**^
Sex (M/F)	241/279	515/1039	**<0.001**^**†**^	351/792	164/247	**<0.001**^**†**^	**<0.001**^**†**^
Median EDSS (IQR)	-	2.0 (1.5;4.5)	**-**	1.5 (1.0;2.5)	6.0 (5.0–6.5)	**-**	<0.001^*^
Median DD (IQR) [y]	-	9.6 (3.4;17.2)	**-**	7.2 (2.5;14.5)	16.6 (10.4;23.7)	**-**	<0.001^*^
DMT (no/MET/HET)	-	461/708/385	**-**	295/598/250	166/110/135	**-**	**<0.001**^**†**^
Mean T2 LV (SD) [mL]	0.2 (0.7)	8.3 (9.9)	<0.001^*^	6.5 (8.0)	13.2 (12.8)	<0.001^**^	<0.001^**^
Mean T2 PV LV (SD) [mL]	-	1.6 (2.2)	**-**	1.2 (1.7)	2.5 (2.9)	**-**	<0.001^*^
Mean T2 JC LV (SD) [mL]	-	2.9 (5.4)	**-**	2.2 (4.1)	4.9 (7.7)	**-**	<0.001^*^
Mean T2 IT LV (SD) [mL]	-	0.2 (0.4)	**-**	0.2 (0.3)	0.4 (0.5)	**-**	<0.001^*^
Mean T2 deep WM LV (SD) [mL]	-	2.6 (3.0)	**-**	2.5 (2.8)	3.0 (3.3)	**-**	0.11*
Mean NcGMV (IQR) [mL]	640 (611;665)	619 (586;649)	<0.001^*^	631 (600;657)	587 (555;614)	<0.001^**^	<0.001^**^
Mean NWMV (IQR) [mL]	723 (705;744)	705 (683;728)	<0.001^*^	707 (688;730)	697 (673;720)	<0.001^**^	<0.001^**^
Mean NThalV (IQR) [mL]	22.6 (21.5;23.5)	20.8 (19.1;22.2)	<0.001^*^	21.3 (19.7;22.6)	19.4 (17.7;20.8)	<0.001^**^	<0.001^**^
Mean NHippV (IQR) [mL]	10.9 (10.3;11.5)	10.3 (9.4;11.1)	<0.001^*^	10.5 (9.7;11.2)	9.6 (8.7;10.4)	<0.001^**^	<0.001^**^
Mean NOtherDGMV (IQR) [mL]	33.6 (31.8;35.2)	31.5 (29.1;33.6)	<0.001^*^	32.2 (29.7;34.1)	29.6 (27.2;31.8)	<0.001^**^	<0.001^**^
Mean NCerGMV (IQR) [mL]	72.4 (65.7;81.4)	67.3 (60.9;75.8)	<0.001^*^	69.1 (61.6;76.7)	63.2 (56.2;70.6)	<0.001^**^	<0.001^**^
Mean NCerWMV (IQR) [mL]	26.7 (24.3;29.3)	24.3 (21.5;27.1)	<0.001^*^	24.8 (22.3;27.5)	22.3 (19.6;25.3)	<0.001^**^	<0.001^**^
Mean NBrainstemV (IQR) [mL]	23.6 (21.9;25.4)	21.7 (20.0;23.6)	<0.001^*^	21.9 (20.2;23.7)	21.0 (19.1;23.0)	<0.001^**^	<0.001^**^

MS patients are first considered as a whole, and then divided according to their clinical phenotype. Significant differences are highlighted in bold. *Mann-Withney U test; ^†^Chi-square test; **post hoc Kruskall-Wallis (Bonferroni corrected).

OSR, IRCCS San Raffaele Scientific Institute; VAN, Università della Campania “Luigi Vanvitelli”; SAP, Sapienza University; SIE, University of Siena; Rel MS, relapsing MS; Prog MS, progressive MS; DD, disease duration; DMT, disease-modifying treatment; MET, medium efficacy treatment; HET, high-efficacy treatment; WM LV, white matter lesion volume; PV, periventricular; JC, juxtacortical; IT, infratentorial; NcGMV, normalized cortical grey matter volume; NWMV, normalized white matter volume; NThalV, normalized thalamic volume; NHippV, normalized hippocampal volume; NOtherDGMV, normalized volume of other deep grey matter nuclei; NcerGMV, normalized cerebellar grey matter volume; NCerWMV, normalized cerebellar white matter volume; NBrainstemV, normalized brainstem volume.

Progressive MS patients showed significantly higher T2 LV than relapsing MS patients across the whole brain and in periventricular (PV), juxtacortical (JC) and infratentorial (IT) compartments (all *p* < 0.001, Table 1), whereas deep WM T2 LV did not differ between these two groups (*p* = 0.11). Progressive MS also had lower brain volumetry compared to relapsing MS patients (all *p* < 0.001).

### 3.2 Classification task: MS patients *vs* HC

**Figure 1:**
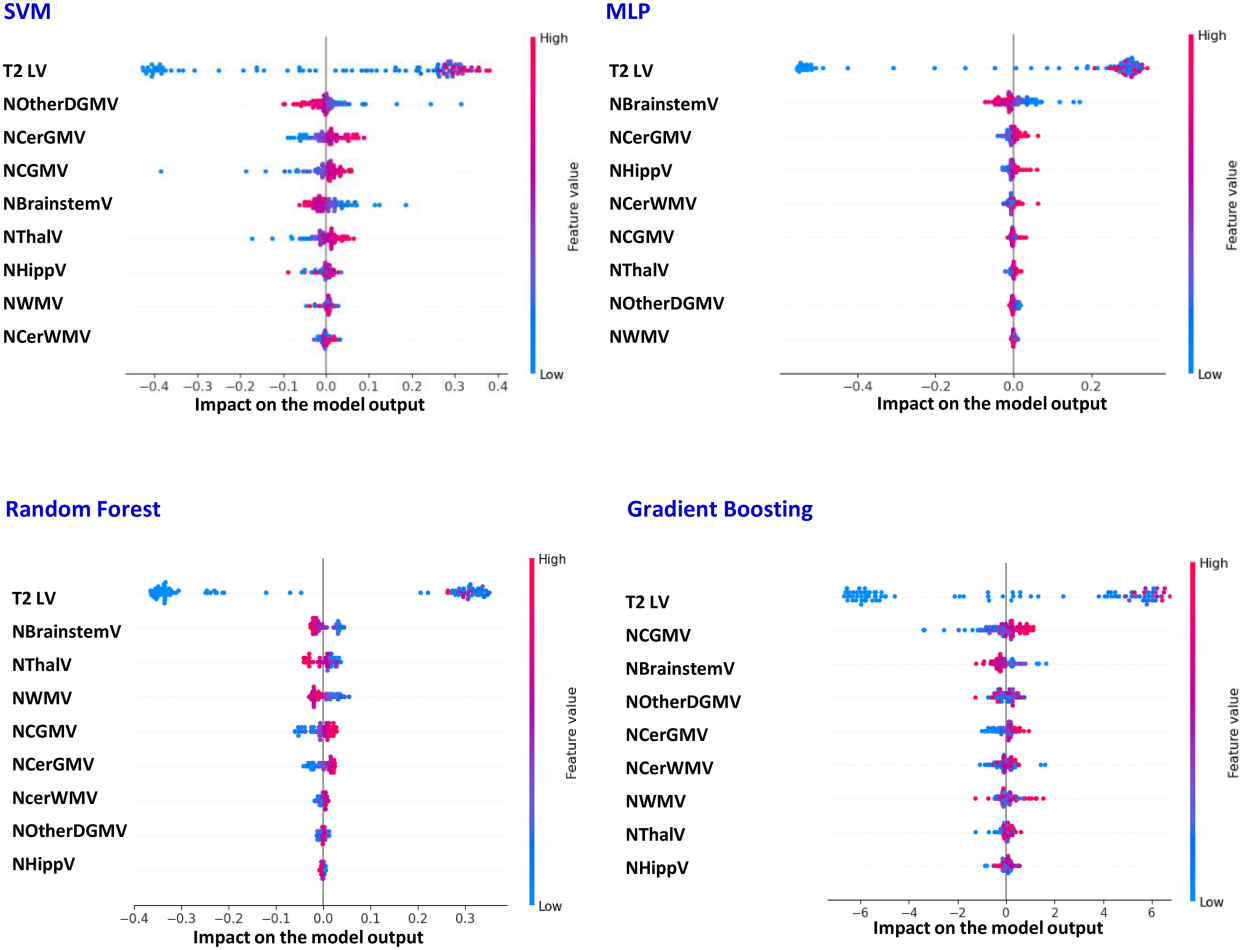
SHAP-based feature contributions to SVM, MLP, Random Forest And Gradient Boosting classification performance (patients vs. healthy controls). Features best explaining the classification performances of support vector machine (SVM), multi-layer perceptron (MLP), Random Forest And Gradient Boosting models when distinguishing patients with multiple sclerosis (MS) from healthy controls (SHAP analysis) using the entire MS cohort. LV, lesion volume; NCGMV, normalized cortical grey matter volume; NWMV, normalized white matter volume; NThalV, normalized thalamic volume; NHippV, normalized hippocampal volume; NOtherDGMV, normalized volume of other deep grey matter nuclei; NCerGMV, normalized volume of cerebellar grey matter; NCerWMV, normalized volume of cerebellar white matter; NBrainstemV, normalized volume of brainstem.

Table 2 reports the average performances of SVM, MLP, Random Forest and Gradient Boosting models in classifying MS patients *vs* HC, first using the entire MS cohort and then related to the two sensitivity analyses described in the Methods. Classification performance was quite high, with accuracies ranging between 89 and 96%, and the area under the receiver operating characteristic curve (AUC-ROC) values between 94 and 98%. The features contributing most to this classification task are illustrated in Figure 1. Although the relative importance of individual measures varied across models, variables consistently ranked among the top contributors included T2 LV, NBrainstemV, NCerGMV and NCGMV. As shown in Supplementary Figure 2, such features were among the top-ranked predictors both in the classification using the full MS cohort and in the analysis limited to early MS patients (*N* = 481). When considering the analysis replacing LV subdivided by topography (Supplementary Figure 3), the most informative locations were the PV and IT compartments, followed by JC T2 LV. Also in this case, NCerGMV and NThalV remained among the most influential variables, with additional (though somewhat more model-dependent) contributions from NCGMV, NBrainstemV and NWMV.

**Table 2:** Average classification performances of support vector machine (SVM), multi-layer perceptron (MLP), Random Forest and Gradient Boosting models when classifying patients with multiple sclerosis (MS) from healthy controls (HC).

Classification task	ML model	Accuracy	Precision	Recall	F1-score	AUC-ROC
All MS patients (*N* = 1,554) *vs* HC (*N* = 520)	SVM	94%	99%	92%	96%	97%
	MLP	96%	99%	96%	97%	98%
	Random Forest	93%	92%	94%	93%	96%
	Gradient Boosting	96%	97%	97%	97%	97%
Sensitivity analysis I: early MS patients (*N* = 481) *vs* HC (*N* = 520)	SVM	93%	95%	91%	93%	97%
	MLP	94%	95%	93%	94%	98%
	Random Forest	93%	92%	94%	93%	96%
	Gradient Boosting	94%	93%	95%	94%	96%
Sensitivity analysis II (including regional LV metrics) on all MS patients (*N* = 1,554) *vs* HC (*N* = 520)	SVM	89%	95%	87%	92%	95%
	MLP	90%	99%	91%	93%	95%
	Random Forest	93%	92%	94%	94%	96%
	Gradient Boosting	89%	92%	94%	93%	94%

The first section of the Table refers to the classification performed on the entire MS cohort, while the second section refers to the classification performed using early MS patients only (disease duration ≤5 years). Finally, the third section refers to the classification task performed using regional lesion volume (LV) metrics. ML, machine learning; AUC-ROC, area under the receiver operating characteristic curve.

The sensitivity analysis performed using models including MRI-derived features only (i.e., excluding demographic and clinical variables) achieved performance levels comparable to those obtained by the integrated dataset (data not shown).

### 3.3 Classification task: relapsing *vs* progressive MS

**Figure 2:**
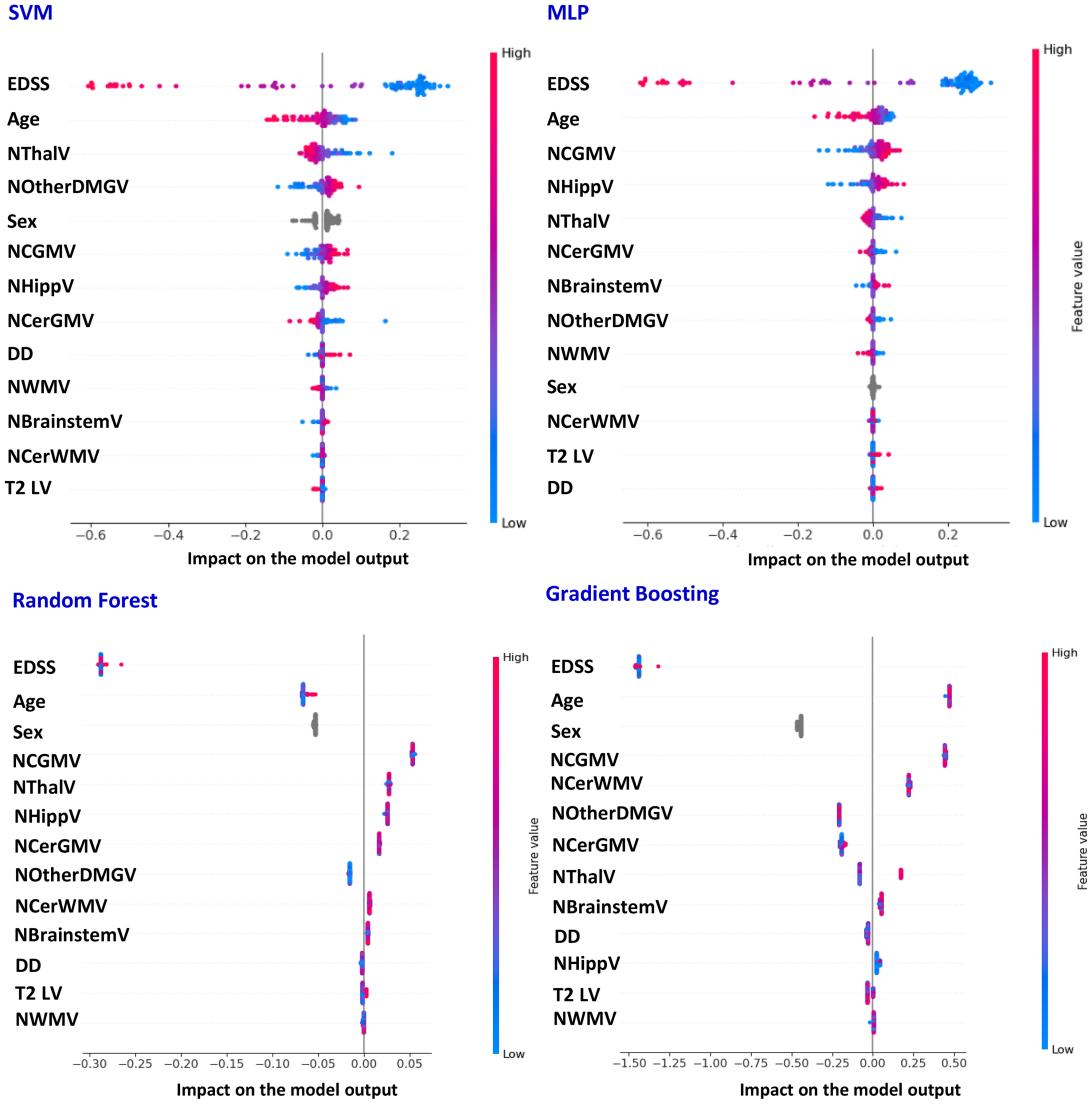
SHAP-based feature contributions to SVM, MLP, Random Forest And Gradient Boosting classification performance (relapsing vs progressive multiple sclerosis). Features best explaining the classification performances of support vector machine (SVM), multi-layer perceptron (MLP), Random Forest And Gradient Boosting models when distinguishing relapsing from progressive patients with multiple sclerosis (MS) (SHAP analysis). EDSS, Expanded Disability Status Scale; LV, lesion volume; NCGMV, normalized cortical grey matter volume; NWMV, normalized white matter volume; NThalV, normalized thalamic volume; NHippV, normalized hippocampal volume; NOtherDGMV, normalized volume of other deep grey matter nuclei; NCerGMV, normalized volume of cerebellar grey matter; NCerWMV, normalized volume of cerebellar white matter; NBrainstemV, normalized volume of brainstem; DD, disease duration.

Table 3 reports the average performances for the classification of relapsing *vs* progressive MS patients, obtained using SVM, MLP, Random Forest and Gradient Boosting models, while the features most contributing to such classification are presented in Figure 2. Also for this task, classification performances were quite high, with all models achieving an accuracy = 92% and AUC-ROC of 96% or 97%. The top variables contributing to classification were consistent across models and included EDSS score, age, NThalV, NCGMV and NHippV.

**Table 3:** Average classification performances of support vector machine (SVM), multi-layer perceptron (MLP), Random Forest and Gradient Boosting models when classifying relapsing- from progressive patients with multiple sclerosis (MS).

Classification task	ML model	Accuracy	Precision	Recall	F1-score	AUC-ROC
Relapsing MS patients (*N* = 1,143) *vs* progressive MS patients (*N* = 411)	SVM	92%	78%	95%	86%	97%
	MLP	92%	83%	89%	86%	97%
	Random Forest	92%	78%	95%	86%	97%
	Gradient Boosting	92%	83%	88%	85%	96%

ML, machine learning; AUC-ROC, area under the receiver operating characteristic curve.

### 3.4 Regression task: EDSS prediction

**Figure 3:**
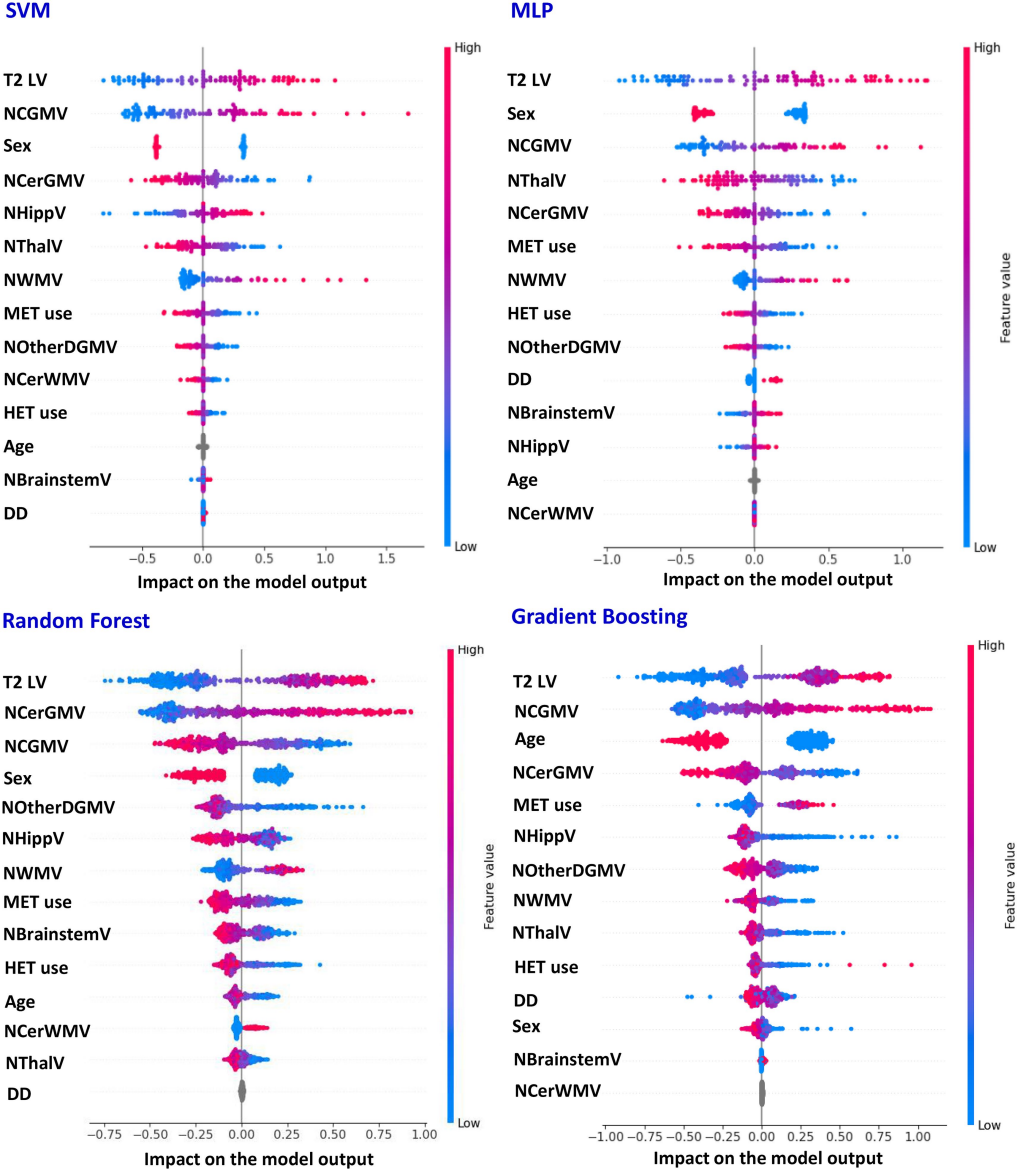
SHAP-derived feature importance in SVM, MLP, Random Forest, and gradient boosting models predicting EDSS. Features best explaining the performances of support vector machine (SVM), multi-layer perceptron (MLP), Random Forest, and gradient boosting models when predicting clinical disability of patients with multiple sclerosis (MS), rated using the Expanded Disability Status Scale (EDSS) score (SHAP analysis). LV, lesion volume; NCGMV, normalized cortical grey matter volume; NWMV, normalized white matter volume; NThalV, normalized thalamic volume; NHippV, normalized hippocampal volume; NOtherDGMV, normalized volume of other deep grey matter nuclei; NCerGMV, normalized volume of cerebellar grey matter; NCerWMV, normalized volume of cerebellar white matter; NBrainstemV, normalized volume of brainstem; DD, disease duration; MET, medium efficacy treatment; HET, high-efficacy treatment.

Table 4 reports the average performances for the prediction of EDSS scores, obtained using SVM, MLP, Random Forest and Gradient Boosting models, while the features most contributing to such prediction are presented in Figure 3. Such ML models were able to predict the EDSS score with an ICC of 0.74 for SVM and 0.76 for MLP. However, the ensemble-based models, Random Forest and Gradient Boosting, showed lower performance, with ICC coefficients of 0.56 for Random Forest and 0.58 for Gradient Boosting. The top variables contributing to EDSS prediction (Figure 3) were T2 LV, male sex and NCGMV, with additional contributions (more dependent upon the model) of NCerGMV, NThalV and NOtherDGMV.

**Table 4:** Average classification performances of support vector machine (SVM), multi-layer perceptron (MLP), Random Forest and Gradient Boosting models when predicting Expanded Disability Status Scale (EDSS) score in patients with multiple sclerosis (MS).

Classification task	ML model	MAE	RMSE	ICC
EDSS prediction	SVM	0.81	1.30	0.74
	MLP	1.18	1.54	0.76
	Random Forest	1.26	1.60	0.56
	Gradient Boosting	1.22	1.56	0.58

ML, machine learning; MAE, mean absolute error; RMSE, root mean square error; ICC, intra-class correlation coefficient.

## 4 Discussion

In this large cross-sectional study, we applied different ML models, integrating demographic, clinical, and MRI features, to perform disease and phenotype classification, as well as patients’ disability prediction. Our results showed that SVM, MLP, Random Forest and Gradient Boosting models achieved strong performance across all tasks, with 89–96% accuracy for classifying MS patients *vs* HC, 92% accuracy for distinguishing relapsing from progressive MS, and an ICC of 0.56–0.76 for clinical disability prediction. Such findings demonstrate the potential of ML-based multiparametric models to enhance understanding of MS pathophysiology at individual level and support their role in advancing personalized MS care. Notably, T2 lesion burden and GM volumetry of key brain structures played a pivotal role in such models, highlighting their relevance as robust neuroimaging biomarkers.

Our findings suggest several interesting implications for clinical practice. First, within our integrated models, quantitative MRI metrics provided relevant information complementing demographic and clinical characteristics to identify unfavorable patients’ profiles. Indeed, SHAP analyses consistently top-ranked quantitative MRI measures among the most influential features across all classification and prediction tasks. This indicates that a systematic quantification of such MRI markers may add clinically meaningful information, potentially more useful than the predominantly qualitative assessments typically reported in routine radiological evaluations. Second, our analysis highlighted the particular relevance of specific CNS structures (especially the brainstem, cerebellum, and thalamus) in relation to disease severity and disability, as atrophy of these regions was strongly associated with unfavorable clinical profiles. This suggests that a quantitative monitoring of these structures may help identifying patients at higher risk of worse outcomes and support more informed prognostic evaluation and follow-up strategies. Finally, our findings may have implications for MRI acquisition protocols in MS, promoting the systematic inclusion of pre-contrast 3D T1-weighted sequences in monitoring protocols, as this sequence enables reliable quantitative assessment of regional brain atrophy, being highly informative for patients’ outcomes.

Our study relied on the use of four ML approaches: SVM, representing a classical ML algorithm; MLP, a neural network (thus a DL model); Random Forest, an ensemble tree-based method based on bagging; and Gradient Boosting, an ensemble boosting technique that sequentially combines weak learners to improve predictive performance (Bonacchi et al., 2022a; Amin et al., 2024; Collorone et al., 2024; Ho, 1995; Hastie et al., 2009; Pilehvari et al., 2024). Remarkably, all these models showed comparable performance across the classification tasks and identified similar sets of top-ranked features. Such an overlap suggests robustness and stability in the underlying data patterns and reinforces the validity of the observed associations. Moreover, the convergence of results between these different modeling paradigms supports the overall reliability and generalizability of our findings, ultimately suggesting that all models can be effectively implemented in clinical research context for classification tasks, with the choice guided by computational resources, dataset size, and domain expertise.

In contrast to the classification tasks, a different pattern emerged for EDSS regression. While SVM and MLP achieved relatively good agreement with the observed scores (ICC = 0.74 and 0.76), the tree-based ensemble methods showed a noticeably lower performance (ICC = 0.56 for Random Forest and 0.58 for Gradient Boosting). One possible explanation is related to the intrinsic properties of these algorithms. Tree-based ensemble methods partition the feature space into discrete regions, which may limit their ability to accurately approximate smooth and continuous relationships between predictors and the EDSS score. In contrast, models such as SVM and MLP can capture more continuous and potentially non-linear mappings, which may be advantageous when modeling clinical disability scales that vary along a continuum. The relatively limited sample size and the presence of noise in clinical measures such as EDSS may further affect the stability of tree-based regression models. Despite these differences in regression performance, the overall consistency observed in feature ranking across models suggests that the main predictive signals in the data remain robust across algorithmic approaches.

SHAP analysis revealed that the most influential variable differentiating MS patients from HC was T2 LV, followed by NCerGMV and (with some model variability) by NBrainstemV and NCGMV. Such findings were observed both in the analysis including all patients and in the sensitivity analysis including early MS patients only, supporting the robustness of such results. The notion that MRI-visible lesions are key discriminators between MS patients and HC is not unexpected and aligns with previous studies (Zhang et al., 2018; Eitel et al., 2019) that already reported high HC/MS patients’ classification accuracy of CNN models trained on conventional MRI images. In addition, we found that cerebellar GM atrophy was another major feature distinguishing HC from MS patients. This is consistent with evidence of extensive cerebellar involvement in MS (Parmar et al., 2018), even at early disease stages. Numerous studies have demonstrated reduced cerebellar GM volume in MS compared with HC (Parmar et al., 2018; Bonacchi et al., 2022b; D'Ambrosio et al., 2017; Parmar et al., 2022). Given the crucial role of the cerebellum in both motor coordination and higher-order functions (Parmar et al., 2018) (including balance, gait control, motor learning, attention, and executive processing), it is plausible that cerebellar atrophy contributes to the distinct neuroanatomical pattern of MS. Although data on brainstem atrophy are limited, previous studies indicated significantly reduced brainstem volume in MS patients *vs* HC (Lee et al., 2018; Ramasamy et al., 2009). Because the brainstem is crucial for motor, sensory, and autonomic processing and serves as a communication hub between the brain, cerebellum, and spinal cord, its atrophy likely plays an important role in differentiating MS from HC. In line with this, previous studies showed a significant reduction of medulla oblongata volume in MS patients compared with HC (Liptak et al., 2008), which was significantly different between MS phenotypes (Sampat et al., 2009), correlated with patients’ clinical characteristics (Liptak et al., 2008; Sampat et al., 2009) and predicted walking impairment (Liptak et al., 2008). Finally, a contribution of cortical GM atrophy to HC/MS classification is not unexpected, since several studies have consistently demonstrated that cortical damage is extensive in MS (Rocca et al., 2017), and often correlates more strongly with disease severity than T2 LV (Rocca et al., 2017), given the key role of cerebral cortex in a wide range of higher-order cognitive and sensorimotor functions.

Interestingly, the sensitivity analysis using topography-based LV metrics showed that PV LV contributed most to classification, followed by IT LV. This likely reflects the early vulnerability of PV regions to MS-related damage, possibly mediated by CSF-related pathogenic factors affecting areas near the ventricular system (Jehna et al., 2015). The second strongest contribution came from IT LV, likely because lesions in this compartment are characteristic of MS (unlike incidental findings in HC) and are known to be associated with poor disease prognosis (Minneboo et al., 2004). Moreover, this region includes cerebellar lesions, which further contribute to the cerebellar damage (atrophy) described above.

When classifying relapsing *vs* progressive MS, the most discriminative features included higher EDSS score, older age, lower NThalV, NCGMV and NHippV. These findings align closely with the well-established clinical and pathological distinctions between MS phenotypes, as progressive MS is typically associated with greater clinical disability and older age (Filippi et al., 2018). The prominence of EDSS and age among the top phenotype predictors therefore reinforces the biological and clinical plausibility of our models, reflecting their ability to capture meaningful markers of disease severity across MS phenotypes. Among the MRI-derived features included in the classification, thalamic atrophy emerged as the most consistent across models. Our results are in line with previous studies showing that thalamic atrophy is already present during the relapsing stages of the disease, but worsens significantly in the progressive phases (Eshaghi et al., 2018; Rocca et al., 2021b). Given the thalamus role as a critical relay hub connecting multiple cortical and subcortical regions (Minagar et al., 2013), its atrophy likely represents a sensitive marker of global neurodegeneration and disease severity, making it a strong MRI-based discriminator between progressive and relapsing MS phenotypes. Finally, as for the HC/MS classification, a role of NCGMV (and to some extent, of NHippV) also emerged in differentiating progressive from relapsing patients. Once again, this is consistent with previous literature showing a marked worsening of cortical and hippocampal atrophy at progressive *vs* relapsing disease stages (Rocca et al., 2021a; Rocca et al., 2017; Eshaghi et al., 2018), driven by increasingly pronounced neurodegeneration, which most likely accounts for the greater severity of this phenotype.

When looking at the regression task predicting clinical disability, we found that the most relevant predictors of EDSS were T2 LV, male sex, NCGMV and NCerGMV, with some additional contribution of NThalV. Our findings are consistent with two previous DL studies, which also reported a significant contribution of GM atrophy to disability prediction in patients with MS. (Coll et al., 2024; Coll et al., 2023) However, our results somewhat extend such observations by suggesting that both WM lesion burden (Filippi et al., 2018) and the degree of GM atrophy in different CNS compartments (Rocca et al., 2017; Eshaghi et al., 2018; Rocca et al., 2021b) play crucial and complementary roles in explaining clinical disability in MS. Altogether, these results highlight that integrating the effects of inflammatory demyelination (captured by T2 LV) and neurodegeneration throughout the CNS (reflected by cortical and cerebellar GM atrophy) provides a more comprehensive framework for understanding disability in MS. Even if evidence in previous literature is somewhat discordant, the identification of male sex as an additional factor associated with greater disability seems to suggest the presence of sex-related differences in disease severity and progression (Ceccarelli, 2022), underscoring the need of deeper investigations of MS-related sex-related disparities.

Although the present study focused on models trained on integrated demographic, clinical, and MRI features, as briefly mentioned in the Results, exploratory analyses using MRI-derived variables alone yielded comparable predictive performance across the investigated tasks. This suggests that MRI features capture a substantial proportion of the relevant predictive signal in our dataset, while the addition of demographic and clinical variables provides only modest incremental improvements. These observations further support the potential utility of MRI-based ML pipelines for scalable and automated clinical applications.

Despite the strengths of this study, including a large, multicenter cohort and harmonized MRI protocols, several limitations should be acknowledged. First, although MRI harmonization was implemented, a slight scanner-related variability might not be entirely excluded. However, as shown in Supplementary Figure 1, we can assume that this potential residual variability is negligible. Second, while the models demonstrated good performance, external validation using independent dataset from different sites or acquisition protocols is necessary to confirm generalizability and to better assess model robustness and clinical applicability beyond the training distribution. This may become feasible with the ongoing expansion of the INNI repository, which is expected to progressively include data from additional centers and acquisition settings. Third, the cross-sectional design limits the ability to capture longitudinal changes in MS patients. Fourth, although potentially clinically relevant, MRI metrics of spinal cord damage could not be included in our models, since these data were not available for the entire study cohort. Finally, to keep our models relatively simple and easily translatable into clinical practice, we did not incorporate non-conventional MRI metrics (such as measures of microstructural damage or functional reorganization). However, the future integration of advanced MRI metrics could further enhance model robustness and completeness and provide a more comprehensive characterization of disease-related changes.

To conclude, our findings demonstrate that ML models can accurately classify MS patients, differentiate clinical phenotypes, and predict disability using integrated demographic, clinical, and MRI data. The consistent identification of lesional and GM volumetric measures as key features across tasks underscores their central role in MS pathology. Our findings suggest that MRI-derived measures, including lesion burden and regional brain volumes, capture a substantial proportion of the predictive signal for MS characterization, phenotype differentiation, and EDSS prediction, with clinical and demographic data providing complementary information that can further refine predictions and complement standard assessment. Ultimately, incorporating these MRI metrics into ML-based decision-support tools holds promise for enhancing personalized care in MS by enabling accurate diagnosis and phenotype classification, and more precise disability prognostication.

## Data Availability

The datasets presented in this study can be found in online repositories. The names of the repository/repositories and accession number(s) can be found at: the anonymized dataset used and analyzed for this study will be published in the San Raffaele Open Research Data Repository (https://ordr.hsr.it/research-data/).

## Footnotes

1
www.inni-ms.org

2
www.xinapse.com

3
https://surfer.nmr.mgh.harvard.edu/
