# Supplementary material for: Machine learning for multiple sclerosis classification and disability prediction using clinical and MRI data
Source: Front Artif Intell. 2026 Apr 10;9:1792870. doi: 10.3389/frai.2026.1792870 (PMC13106177; doi:10.3389/frai.2026.1792870)
Supplement: Supplementary file 1 [file Data_sheet_1.pdf]

Supplementary Table 1. Parameters used for the acquisition of MRI sequences at the four participating centres.

	Milan		Naples		Rome	Siena
Scanner	Philips Achieva	Philips Ingenia	GE Signa HDxt	GE DV750	Siemens Magnetom Verio	Philips Achieva
Sequence for WM lesion assessment	DE TSE:<br>TR=range 2599-2910 ms<br>TE=16/80 ms<br>FA=90°<br>matrix=256x256<br>FOV=240x240 mm<br>thickness=3 mm<br>n. of slices=range 44-50<br>orientation=axial	3D FLAIR:<br>TR=4800 ms<br>TE=270 ms<br>TI=1650 ms<br>matrix=256×256<br>FOV=256×256 mm<br>thickness=1 mm<br>n. of slices=192<br>orientation=sagittal	DE TSE:<br>TR=3080 ms<br>TE=24/127.5 ms<br>FA=90°<br>matrix=256x384<br>FOV=240x240 mm<br>thickness=3 mm<br>n. of slices=44<br>orientation=axial	DE TSE:<br>TR=5297 ms<br>TE=18/93<br>FA=110°<br>matrix=384x384<br>FOV=240x240 mm<br>thickness=3 mm<br>n. of slices=44<br>orientation=axial	DE TSE:<br>TR=range 3320-5310 ms<br>TE=10/103 ms<br>FA=150°<br>matrix=384x384<br>FOV=220x220 mm<br>thickness=range 3-4 mm<br>n. of slices=range 40-45<br>orientation=axial	DE TSE:<br>TR=4000 ms<br>TE=15/100 ms<br>FA=90°<br>matrix=240x240 (recon 352x352)<br>FOV=240x240 mm<br>thickness=3 mm<br>n. of slices=44<br>orientation=axial
3D T1-weighted	TR=25 ms<br>TE=4.6 ms<br>FA=30°<br>matrix=256x256<br>FOV=230x230x176 mm<br>thickness=0.8 mm<br>n. of slices=220<br>orientation=axial	TR=7.0 ms<br>TE=3.2 ms<br>TI=1000 ms<br>FA=8°<br>matrix=256x256<br>FOV=256x256x204 mm<br>thickness=1 mm<br>n. of slices=204<br>orientation=sagittal	TR=6.98 ms<br>TE=2.85 ms<br>TI=650 ms<br>FA=8°<br>matrix=256x256<br>FOV=256x256x199.2 mm<br>thickness=1.2 mm<br>n. of slices=166<br>orientation=sagittal	TR=6.9 ms<br>TE=2.99 ms<br>TI=650 ms<br>FA=9°<br>matrix=256x256<br>FOV=256x256x176 mm<br>thickness=1 mm<br>n. of slices=176<br>orientation=sagittal	TR=1900 ms<br>TE=2.9 ms<br>TI=900 ms<br>FA=9°<br>matrix=256x256<br>FOV=256x256x176 mm<br>thickness=1 mm<br>n. of slices=176<br>orientation=sagittal	TR=10 ms<br>TE=3.9 ms<br>TI=900 ms<br>FA=8°<br>matrix=256x256<br>FOV=256x256x192 mm<br>thickness=1 mm<br>n. of slices=192<br>orientation=axial

Abbreviations: DE TSE=dual echo turbo spin echo; FLAIR=fluid attenuated inversion recovery; WM=white matter; TR=repetition time; TE=echo time; TI=inversion time; FA=flip angle; FOV=field of view.

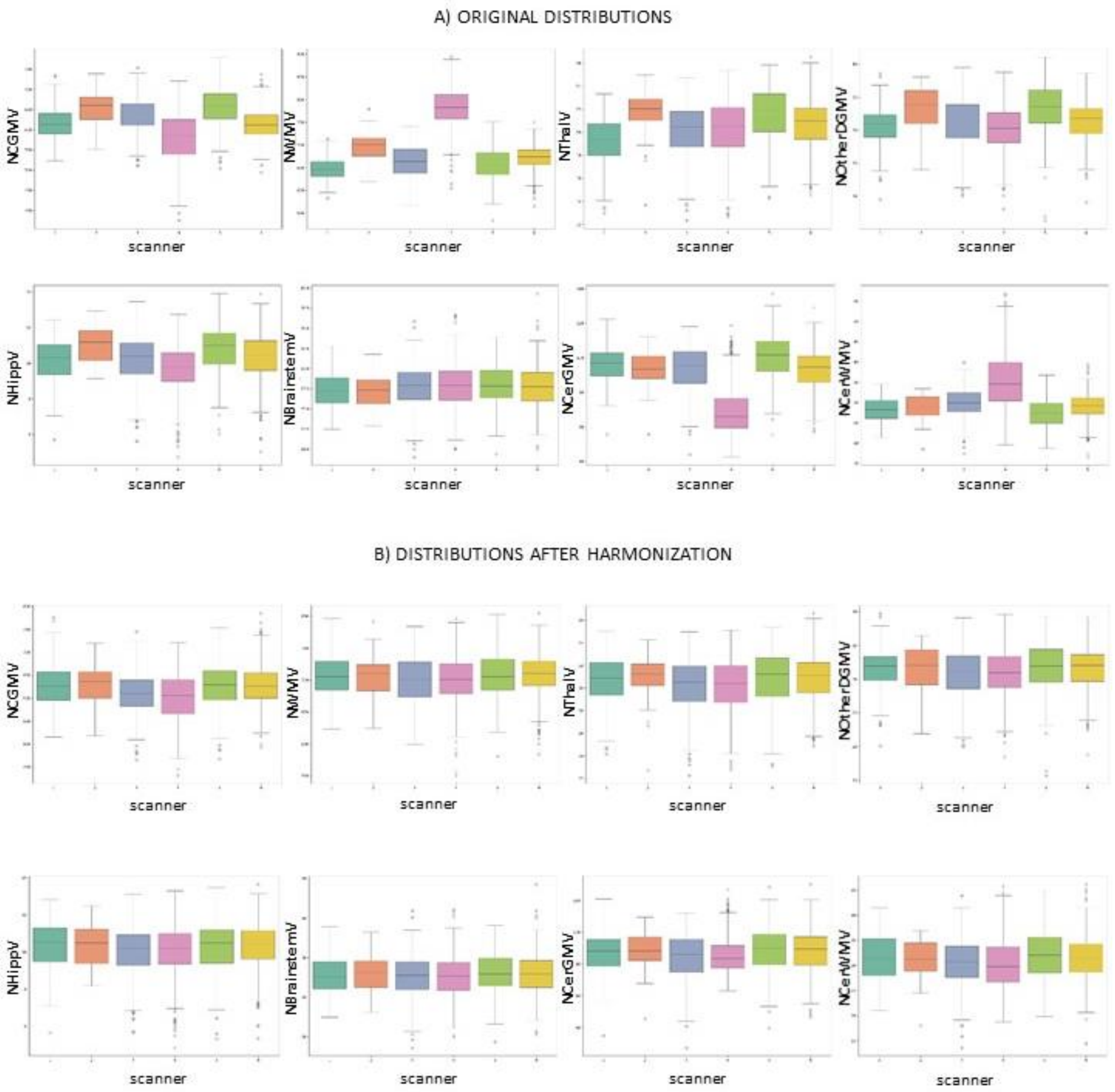

Supplementary Figure 1. Boxplots showing the distributions of MRI measures across scanners (scanner 1=Philips Achieva, University of Siena; scanner 2=GE Signa HDxt, University of Campania “Luigi Vanvitelli”; scanner 3=Philips Achieva, IRCCS San Raffaele Scientific Institute; scanner 4=Philips Ingenia, IRCCS San Raffaele Scientific Institute; scanner 5=GE DV750, University of Campania “Luigi Vanvitelli”; scanner 6=Siemens MagnetomVerio, Sapienza University of Rome) before (panel A) and after (panel B) harmonization performed using NeuroCombatprocedure. Abbreviations: NCGMV=normalized cortical grey matter volume; NWMV=normalized white matter volume; NThalV=normalized thalamic volume; NOtherDGMV=normalized volume of other deep grey matter nuclei; NHippV=normalized hippocampal volume; NBrainstemV=normalized brainstem volume; NCerGMV=normalized cerebellar grey matter volume; NCerWMV=normalized cerebellar white matter volume.

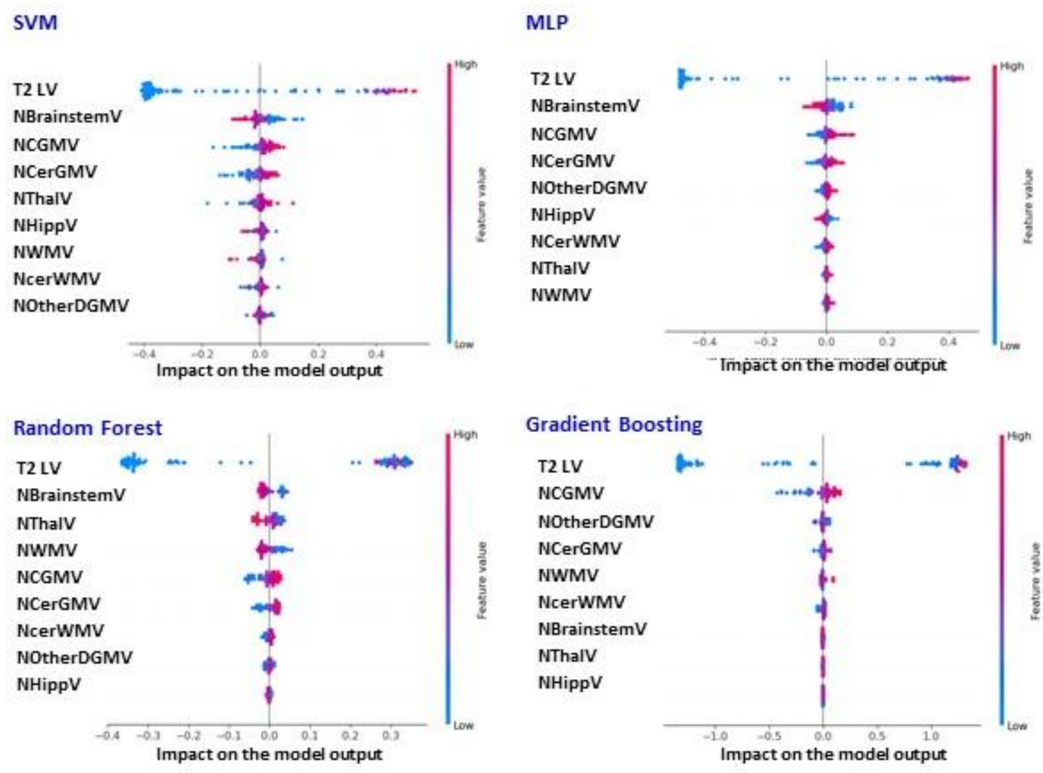

Supplementary Figure 2. SHAP-based feature contributions to SVM, MLP, Random Forest e Gradient Boosting classification performance (patients vs healthy controls) – Sensitivity Analysis I. Features best explaining the classification performances of support vector machine (SVM), multi-layer perceptron (MLP), Random Forest and Gradient Boosting models when distinguishing patients with multiple sclerosis (MS) from healthy controls (SHAP analysis) performed using only early MS patients (disease duration ≤5 years, sensitivity analysis I). Abbreviations: LV=lesion volume; NCGMV=normalized cortical grey matter volume; NWMV=normalized white matter volume; NThalV=normalized thalamic volume; NHippV=normalized hippocampal volume; NOtherDGMV=normalized volume of other deep grey matter nuclei; NCerGMV=normalized volume of cerebellar grey matter; NCerWMV=normalized volume of cerebellar white matter; NBrainstemV=normalized volume of brainstem.

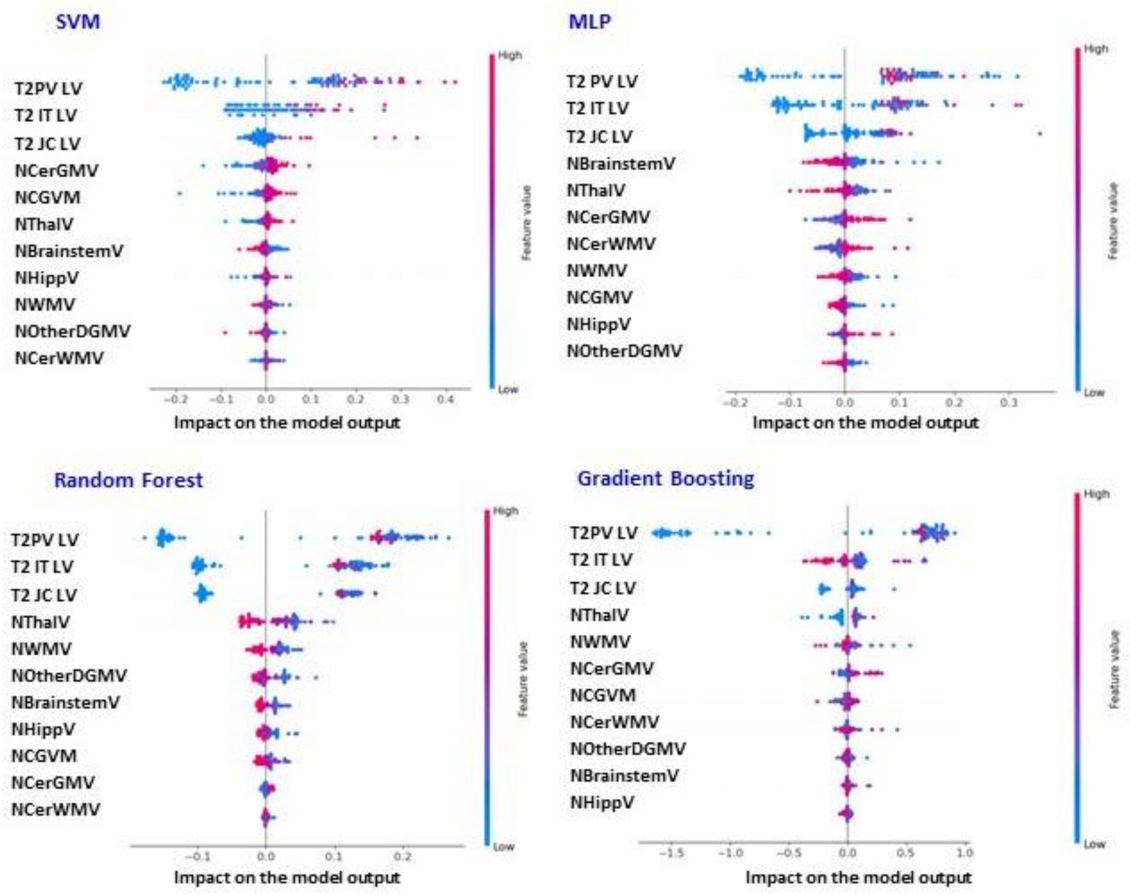

Supplementary Figure 3. SHAP-based feature contributions to SVM, MLP, Random Forest e Gradient Boosting classification performance (patients vs healthy controls) – Sensitivity Analysis II. Features best explaining the classification performances of support vector machine (SVM), multi-layer perceptron (MLP), Random Forest and Gradient Boosting models when distinguishing patients with multiple sclerosis (MS) from healthy controls (SHAP analysis) performed using regional lesion volume metrics (sensitivity analysis II).

Abbreviations: LV=lesion volume; PV=periventricular; JC=juxta-cortical; IT=infratentorial; NCGMV=normalized cortical grey matter volume; NWMV=normalized white matter volume; NThalV=normalized thalamic volume; NHippV=normalized hippocampal volume; NOtherDGMV=normalized volume of other deep grey matter nuclei; NCerGMV=normalized volume of cerebellar grey matter; NCerWMV=normalized volume of cerebellar white matter; NBrainstemV=normalized volume of brainstem.
